# Circular RNA CREBBP modulates cartilage degradation by activating the Smad1/5 pathway through the TGFβ2/ALK1 axis

**DOI:** 10.1038/s12276-022-00865-2

**Published:** 2022-10-12

**Authors:** Yiyang Xu, Guping Mao, Dianbo Long, Zengfa Deng, Ruobin Xin, Ziji Zhang, Ting Xue, Weiming Liao, Jie Xu, Yan Kang

**Affiliations:** 1 Department of Joint Surgery, The First Affiliated Hospital, Sun Yat-sen University, 510080 Guangzhou, Guangdong Province China; 2 Guangdong Provincial Key Laboratory of Orthopaedics and Traumatology, 510080 Guangzhou, Guangdong Province China; 3 Department of Orthopaedics, Fujian Provincial Hospital, Shengli Clinical Medical College, Fujian Medical University, 350001 Fuzhou, Fujian Province China; 4 Fujian Provincial Hospital South Branch, Center of Health Management, Shengli Clinical Medical College of Fujian Medical University, Fuzhou, China

**Keywords:** Long non-coding RNAs, Phosphorylation

## Abstract

Osteoarthritis, characterized by articular cartilage degradation, is the leading cause of chronic disability in older adults. Studies have indicated that circular RNAs are crucial regulators of chondrocyte development and are involved in the progression of osteoarthritis. In this study, we investigated the function and mechanism of a circular RNA and its potential for osteoarthritis therapy. The expression levels of circCREBBP, screened by circular RNA sequencing during chondrogenic differentiation in adipose tissue-derived stem cells, and TGFβ2 were significantly increased in the cartilage of patients with osteoarthritis and IL-1β-induced chondrocytes. circCREBBP knockdown increased anabolism in the extracellular matrix and inhibited chondrocyte degeneration, whereas circCREBBP overexpression led to the opposite effects. Luciferase reporter assays, rescue experiments, RNA immunoprecipitation, and RNA pulldown assays confirmed that circCREBBP upregulated TGFβ2 expression by sponging miR-1208, resulting in significantly enhanced phosphorylation of Smad1/5 in chondrocytes. Moreover, intra-articular injection of adeno-associated virus-sh-circCrebbp alleviated osteoarthritis in a mouse model of destabilization of the medial meniscus. Our findings reveal a critical role for circCREBBP in the progression of osteoarthritis and provide a potential target for osteoarthritis therapy.

## Osteoarthritis: Circular RNA linked to cartilage degeneration

A circular RNA implicated in cartilage degeneration could offer a new therapeutic target for treating osteoarthritis. Yan Kang from Sun Yat-sen University, Jie Xu from Fujian Provincial Hospital, and colleagues in China found that a circular RNA called circCREBBP is highly expressed in cartilage samples taken from the knee joints of individuals with osteoarthritis. Working with cartilage precursor cells, the researchers showed that the circular RNA induces destruction of the surrounding extracellular matrix through its inhibitory effects on a microRNA. This microRNA normally blocks expression of a growth factor involved in matrix degradation. When bound to circCREBBP, the microRNA can no longer keep the growth factor in check and osteoarthritis results. Inhibiting circCREBBP activity prevented cartilage breakdown and alleviated osteoarthritis in mouse models, suggesting that a similar therapeutic strategy could work in humans.

## Introduction

Osteoarthritis (OA) is a well-known age-related disease affecting people worldwide, causing severe impairment of joint function and limiting the quality of life of patients^1,2^. Once OA has reached the progressive and end stages, the disease spreads throughout the joint, leading to pain, stiffness, and loss of normal joint function^3,4^. Alterations in cartilage function appear to be driven by an imbalance in the anabolic and catabolic activities of the extracellular matrix (ECM) in chondrocytes^5–7^. Thus, reversing the imbalanced homeostasis in the ECM of chondrocytes is a core goal of OA therapy^8^. Studies on the cellular regulation of chondrogenic differentiation of mesenchymal stem cells, particularly adipose-derived stem cells (ADSCs), may enhance the understanding of the etiology and pathogenesis of OA and promote cartilage regeneration in the clinic. Increasing evidence suggests that mesenchymal stem cell-derived molecules play a vital role in modulating chondrogenesis, matrix synthesis, and cell aging^9,10^.

Circular RNAs (circRNAs) are characterized by the presence of a covalent bond linking the 3′ and 5′ ends and are generated by backsplicing^11,12^. When circRNAs were first discovered 40 years ago, they were believed to be the products of a splicing error and to not have important roles^13,14^. However, with the advent of next-generation sequencing, the number of published studies on circRNA biology and functions has greatly increased; these studies have demonstrated that circRNAs are important for cellular differentiation and tissue homeostasis as well as in disease progression^15–17^. Recently, Yang et al. found that circCREBBP (hsa_circ_0007637) upregulated the expression of LEFTY2, arrested the cell cycle, and inhibited proliferation in LX-2 cells, which eventually led to alleviation of liver fibrosis^18^. Qiao et al. revealed that circCREBBP (hsa_circ_0037655) served as an oncogene by promoting cell viability and invasion through phosphoinositide 3-kinase (PI3K) signaling^19^. Zhang et al. revealed that circCREBBP could promote glioma cell growth, migration, invasion, and glutamine metabolism and impede tumor growth in nude mice^20^. Although the potential role of CREBBP-associated circular transcripts in skeletal muscle during chicken embryonic development has been demonstrated, its effect on human chondrogenesis and cartilage degeneration remains unknown^21^.

The transforming growth factor β (TGFβ) superfamily, which includes TGFβs (TGFβ1, TGFβ2, and TGFβ3), bone morphogenic proteins (BMPs), activins, and Nodal, plays crucial roles in cell proliferation, differentiation, organic development, ECM remodeling, and tissue homeostasis^22,23^. The signaling specificity of the TGFβ pathway is determined by type I (activin receptor-like kinases 1–7 [ALK1–7]) and type II receptors (TGFβRII, ActR-II, ActR-IIB, BMP receptor II [BMPR-II], and AMHR-II). In humans, the Smad family contains eight members, five of which (Smads 1, 2, 3, 5, and 8), called receptor-regulated Smads, have a C-terminal Ser-X-Ser motif for phosphorylation^24,25^. TGFβ-related pathways involve heteromeric complexes of two type I receptors and two type II receptors, which activate members of the Smad family of signal transducers^24^. Thus, after phosphorylation by the type II receptor, type I receptor kinases recruit and phosphorylate Smad proteins, which are translocated into the nucleus to regulate gene expression. TGFβs, specifically TGFβ1 and TGFβ3, appear to bind type II receptors with high affinity, which then recruit the lower-affinity type I receptor. In contrast, BMPs bind both type I and type II receptors with equal affinity and greater flexibility^26,27^. Upon TGFβ2-specific knockdown, rather than TGFβ1 or TGFβ3 knockdown, the RNA expression of collagen II was induced^28^. However, the molecular functions and mechanisms of action of TGFβ2 in chondrogenesis and cartilage degradation remain unclear.

In this study, we investigated the functions of circCREBBP in cartilage degradation by analyzing the RNA sequences to profile circular transcript expression during chondrogenic differentiation in ADSCs, among various other experimental approaches.

## Materials and methods

### Sample collection, cell isolation and culture, and chondrogenesis

This study was approved by the First Affiliated Hospital of Sun Yat-sen University Clinical Research Ethics Committee ([2013]C-110), and the experiments were performed in accordance with approved guidelines. Written informed consent was obtained from all subjects. Adipose specimens were acquired from 12 healthy young adults who underwent abdominal liposuction (mean age, 28 years; range, 24–31 years; female, *n* = 12) at the First Affiliated Hospital of Sun Yat-sen University. ADSCs were isolated using collagenase type I (C0130, Sigma-Aldrich, St. Louis, MO, USA) as described previously^29^ and cultured in basal medium (alpha-modified Eagle’s medium (α-MEM); Gibco Life Technologies, Grand Island, NY, USA) with 10% fetal bovine serum (FBS). Chondrogenesis was induced in ADSCs on the third passage by micromass culture, as previously reported^9^. Briefly, ADSCs were resuspended at 2 × 10^7^ cells/mL in incomplete chondrogenic medium (97 mL of human adipose stem cell chondrogenic differentiation basal medium, 10 μL of dexamethasone, 300 μL of ascorbate, 1 mL of ITS (insulin, transferrin, selenium) supplement, 100 μL of sodium pyruvate, and 100 μL of proline; Cyagen Biosciences, Guangzhou, China). Droplets of resuspended cells (12.5 μL) were carefully transferred to the individual wells of a 24-well plate and incubated at 37 °C for 90 min. Concentrated droplets were cultured in 500 μL of complete chondrogenic induction medium and prepared by addition of 20 ng transforming growth factor β3 (TGFβ3) to 1 mL of incomplete chondrogenic medium (Cyagen Biosciences). Samples were collected for experiments at 0, 3, 14, and 21 days during ADSC chondrogenesis.

Osteoarthritic cartilage samples were acquired from the knee joints of 24 patients (6 males, 18 females; average age: 67 years) during total knee replacement operations. Normal cartilage samples were collected from the hip joints of 12 patients (6 males, 6 females; mean age: 60 years) without a previous history of OA or rheumatoid arthritis during total hip replacement surgeries because of femoral neck fracture. The cartilage specimens were dissected away from the subchondral bone and cut into 5 × 10-mm pieces. The pieces of cartilage were successively digested with 4 mg/mL protease (P5147, Sigma-Aldrich) for 90 min and 0.25 mg/mL collagenase P (11213873001, Roche, Mannheim Germany) for 12 h as described previously^30^. Chondrocytes were cultured in Dulbecco’s modified Eagle’s medium/F-12 (Gibco Life Technologies) containing 10% FBS and 1% penicillin and streptomycin (Gibco Life Technologies). The chondrocytes were used in the experiments within one week and without passaging to avoid dedifferentiation.

### Cell lines

The HEK293T and SW1353 human chondrosarcoma cell lines were obtained from the National Collection of Authenticated Cell Cultures (Shanghai, China). ATDC5 mouse cells were purchased from the American Type Culture Collection (Manassas, VA, USA). HEK293T cells and SW1353 cells were maintained in Dulbecco’s modified Eagle’s medium with 10% FBS (Gibco Life Technology) and 1% penicillin and streptomycin. ATDC5 cells were cultured in α-MEM containing 5% FBS and 1% penicillin and streptomycin. All cells were cultured in an incubator at 37 °C with 5% CO_2_.

### RNA extraction, reverse transcription, and quantitative real-time PCR analysis

RNA extraction and reverse transcription were performed as described in our previous study^31^. Briefly, total RNA was extracted from the isolated chondrocytes using the miRNA Mini Kit (Qiagen, Hilden, Germany) according to the manufacturer’s protocol. Nuclear and cytoplasmic RNA were extracted following the instructions of the Nuclear and Cytoplasmic Extraction Reagents Kit (Beyotime Biotechnology, Beijing, China). RT‒qPCR was performed using an ABI ViiA™ 7 Real-Time PCR System (Applied Biosystems, Foster City, CA, USA). The specific primers used for these analyses are listed in Supplementary Table 1. Gene expression was calculated using the 2^-ΔΔCt^ method^32^.

### Western blotting

Western blot analysis was performed as described previously^10^. Briefly, the total cellular protein was isolated from human and mouse chondrocytes and SW1353 cells using radioimmunoprecipitation assay lysis buffer (Beyotime Biotechnology) containing protease inhibitors (1:100, Abcam, Cambridge, UK). Electrophoresis was started at 80 V for 20 min and then continued using 120 V for 1 h, followed by 250 mA transfer for 1.5 h. After blocking for 15 min with protein-free rapid blocking buffer (PS108, Epizyme, Shanghai, China), the membranes were incubated overnight at 4 °C with primary antibodies (Supplementary Table 2). Following incubation, the membranes were treated with the corresponding horseradish peroxidase-conjugated secondary antibodies (1:3000, Cell Signaling Technology, Danvers, MA, USA) at 20–25 °C for 1 h. The protein bands were detected using a ChemiDoc Touch (Bio-Rad Laboratories, Hercules, CA, USA) and analyzed using Image Lab^TM^ (Bio-Rad Laboratories). The intensity of bands was compared using ImageJ software (NIH, Bethesda, MD, USA).

### Immunofluorescence analysis

Immunofluorescence (IF) analysis of the transfected cells (after 72 h) was performed as previously reported^33^. After deparaffinization and rehydration, the sections were blocked in 5% bovine serum albumin (BSA) buffer for 30 min. The sections were then incubated with primary antibodies overnight at 4 °C (Supplementary Table 2). Normal rabbit IgG was used instead of a primary antibody on the NC section, and the secondary antibody was conjugated to goat anti-rabbit IgG for 50 min at 24–26 °C. After incubation, the sections were stained with DAPI and mounted with an antifade mounting medium. Images were captured using a confocal laser microscope (LSM 780, Zeiss, Oberkochen, Germany) with a ×40 objective lens.

### Transfection

Transfection was performed using Lipofectamine 3000 (Thermo Fisher Scientific, Waltham, MA, USA) according to the manufacturer’s instructions. Cells were transfected with miR-1208 mimics, miR-mimic-control, circCREBBP siRNAs, TGFβ2 siRNAs, or si-NC (RiboBio, Guangzhou, China) at 50 nM, whereas miR-1208 inhibitor or inhibitor control (RiboBio) was used at a concentration of 100 nM. The overexpression vector pLC5-circCREBBP was constructed using amplified DNA fragments, including the sequence of full-length human circCREBBP_007 cDNA (713 bp, NM_004380) obtained from Geneseed (Guangzhou, China). Human pGenesil-1-si-h-circCREBBP based on valid siRNA-circCREBBP sequences was purchased from HanBio (Shanghai, China). The specific sequences are listed in Supplementary Table 3. Specific inhibitors of ALK5 (SB-505124, HY-13521, 1 μM) and ALK1 (K02288, HY-12278, 0.5 µM) were purchased from MedChemExpress (Monmouth Junction, NJ, USA).

### Dual-luciferase constructs and reporter assay

For analysis of circCREBBP targeting by miR-1208, the putative miR-1208 complementary site in the 3′Untranslated Region (UTR) of circCREBBP or its mutant sequence was cloned into the pMIR-REPORT Luciferase-hsa_circCREBBP vector (Obio Technology, Shanghai, China). The resultant vectors were cotransfected into HEK293T cells with miR-1208 mimics or negative control (NC). pSI-REPORT Luciferase-h-TGFβ2-3′UTR-WT and pSI-REPORT Luciferase-h-TGFβ2-3′UTR-MT were constructed similarly (HanBio).

HEK293T cells were plated in 96-well plates at 7–8 × 10^3^ cells per well and cotransfected with 0.2-μg reporter plasmid and 50 nM miR-1208 mimics or miR-NC using Opti-MEM (Invitrogen, Carlsbad, CA, USA) and Lipofectamine 3000. Luciferase activity was measured using the Dual-Luciferase Reporter Assay System (Promega, Madison, WI, USA) after 48 h. The optical density of the resulting solution was measured using a Synergy H1MF microplate reader (BioTek, Winooski, VT, USA).

### RNA immunoprecipitation assay

Ago2-RIP experiments were performed using the Magna RIP RNA-Binding Protein Immunoprecipitation Kit (Millipore, Billerica, MA, USA). Resuspended RNA complexes were immunoprecipitated overnight at 4 °C with anti-Ago2 antibody (1:50, #ab186733, Abcam) or rabbit IgG-coated magnetic beads included in the kit. Absorbed RNAs were treated with proteinase K buffer and purified with TRIzol reagent for cDNA synthesis. RT‒qPCR was used to detect specific RNA expression using the sequence-specific primers mentioned above.

### RNA fluorescence in situ hybridization

FAM (488)-labeled circCREBBP probes were designed and synthesized by Servicebio (Wuhan, China). Slides containing human chondrocytes or SW1353 cells were fixed with 4% paraformaldehyde for 20 min. After prehybridization using 1× PBS and 0.5% Triton X-100 for 1 h, hybridization was performed overnight with specific probes at 42 °C. The slides were washed three times using 2×, 1×, and 0.5× saline-sodium citrate buffer. Images were acquired using a confocal microscope (Zeiss LSM710). Nuclei were counterstained with 2 mg/mL DAPI for 8 min at 20–25 °C. The specific sequences of the probes are listed in Supplementary Table 3.

### Histological analyses

Cartilage specimens were fixed in 4% paraformaldehyde (Sigma-Aldrich), decalcified, embedded in paraffin, and cut into 5-μm-thick sections. Prior to analysis, the sections were deparaffinized, rehydrated, and then stained with 0.1% safranin-O solution and 0.001% fast green solution (Sigma-Aldrich). Collagen II, aggrecan, MMP13, and TGFβ2 expression was analyzed by IHC as described previously^30^.

For in situ hybridization of circCREBBP, circCrebbp, and miR-1208 in ADSC microspheres and cartilage tissues, specific RNA probes (Servicebio) were used as described in our previous study^30^. The specific sequences of the probes are shown in Supplementary Table 3.

### Destabilization of the medial meniscus mouse model of OA

The animal use protocol described below was reviewed and approved by the Institutional Animal Care and Use Committee of Sun Yat-Sen University (SYSU-IACUC-2020-000487). Thirty-six male WT C57BL/6J mice (purchased from GemPharmatech, Co., Jiangsu, China) were housed under specific pathogen-free conditions and used in experiments at 8 weeks of age. The mice were provided with a normal diet and had access to water ad libitum.

Twelve mice were randomly divided into two groups (control and TGFβ2 injections). Recombinant human TGFβ2 (200 ng; PeproTech, Rocky Hill, NJ, USA) dissolved in 10 μL of physiological saline (0.9% NaCl) + 0.1% ultrapure BSA (Sigma-Aldrich) was injected into the left knee cavity once per week for a month in the TGFβ2 injection group, and an equal volume of saline + 0.1% BSA was administered to the control group.

The other 24 mice were anesthetized by 2–3% isoflurane inhalation followed by 1.5–2% isoflurane for anesthesia maintenance. The adeno-associated virus (AAV) shRNA-circCrebbp and AAV-control were constructed and packaged by HanBio. For induction of OA in vivo, the mice were subjected to destabilization of the medial meniscus (DMM) surgery of the left knee. After 4 weeks, all mice were randomly divided into four cohorts (*n* = 6/cohort): sham, DMM + AAV-control, DMM + AAV-shRNA-circCrebbp, and DMM + AAV-shRNA-circCrebbp+TGFβ2. Mice from the latter three cohorts were administered multiple intra-articular injections of 15 μL of AAV-control, AAV-shRNA-circCrebbp, and AAV-shRNA-circCrebbp and TGFβ2 once per week for 4 weeks. One week after the last injection, the knee joints of the mice were obtained, and further histochemical analysis was performed.

### Statistical analyses

All experiments were performed in at least three biological replicates. Experimental data are shown as the means ± standard deviations. Statistical analysis was performed by unpaired two-tailed Student’s *t* test between two groups. Multiple group comparisons were performed by one-way analysis of variance (ANOVA) or Kruskal–Wallis test followed by a Bonferroni or Dunn post hoc test. Differences were considered statistically significant at *P* values lower than 0.05. Data analysis was performed using SPSS 22.0 software (SPSS, Inc., Chicago, IL, USA).

## Results

### Expression of the circular transcript of CREBBP increases until day 14 during chondrogenesis in ADSCs

**Fig. 1:**
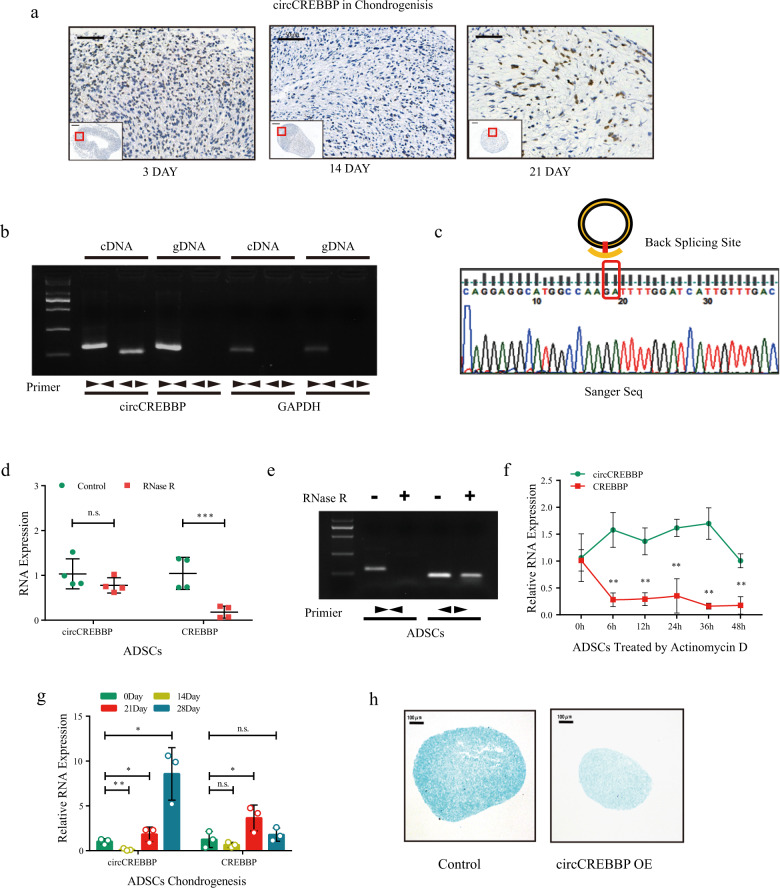
Characterization of circCREBBP during the chondrogenesis of ADSCs. **a** In situ hybridization of circCREBBP-labeled in the pathological section of microspheres during chondrogenesis. **b** RT‒qPCR products generated with divergent primers and linear primers in ADSCs showing circularization of circCREBBP. cDNA, complementary DNA. gDNA, genomic DNA. **c** The presence of circCREBBP was validated by Sanger sequencing. The red box represents specific head-to-tail splicing sites of circCREBBP. **d** circCREBBP and CREBBP expression in ADSCs treated with or without RNase R was detected by RT‒qPCR (*n* = 4). **e** Agarose gel electrophoresis assay for PCR products from divergent primers and linear primers in ADSCs treated with or without RNase R. **f** circCREBBP and CREBBP expression in ADSCs treated with actinomycin D for different durations was detected by RT‒qPCR (*n* = 3). **g** circCREBBP and CREBBP expression during chondrogenesis of ADSCs assessed by RT‒qPCR (*n* = 3). **h** ADSCs were transfected with circCREBBP-OE or control plasmid and then induced to undergo chondrogenesis in 3D culture for 14 days. Alcian blue staining was performed in the pathological section of chondrogenesis microspheres. GAPDH was used as the internal reference. Two-tailed Student’s *t* test or one-way analyses of variance were used for a significance level, and Dunnett’s test was used to perform multiple comparisons. Each bar represents the mean ± SD. *P* < 0.05, *P* < 0.01, *P* < 0.001, n.s. no significance.

We performed RNA-Seq with a circRNA microarray (NCBI BioProject: PRJNA755468) to determine the circRNA expression patterns during chondrogenesis in ADSCs. The differentially expressed circRNAs from all three paired samples, including undifferentiated and chondro-differentiated ADSCs, are shown in a heatmap (Supplementary Fig. 1a). The threshold we used to screen significantly up- or downregulated circRNAs was length <1000 nucleotides (nt), fold change >2.0 and FDR < 0.05. We focused on circCREBBP, also called circ0007637, which was found to have the largest number of Ago2-binding sites among circRNAs according to the CircleInteractome database and is significantly differentially expressed during chondrogenesis in ADSCs (Supplementary Fig. 1b). In the pathological sections of microspheres observed by in situ hybridization analysis on days 0, 14, and 21, circCREBBP expression showed the lowest level on day 14 (Fig. 1a).

To validate the specific head-to-tail characteristics of circular RNA, we first designed convergent primers to amplify CREBBP mRNA and divergent primers to amplify circCREBBP in undifferentiated ADSCs. Gel electrophoresis of the amplified products revealed that circCREBBP could only be amplified from cDNA rather than from genomic DNA (Fig. 1b). In subsequent steps, the head-to-tail splicing junction site was confirmed by Sanger sequencing (Fig. 1c). Because of the high stability of the circular structure, we further tested the sensitivities of circCREBBP and CREBBP mRNA to RNase R. Both real-time quantitative PCR (RT‒qPCR) analysis of cDNA generated from RNase R-treated RNAs and subsequent gel electrophoresis showed notable resistance to RNase R digestion (Fig. 1d, e). In addition, the half-life of circCREBBP was longer than that of CREBBP mRNA after treatment with actinomycin D (Fig. 1f). Furthermore, circCREBBP expression temporarily decreased before day 14 of chondrogenesis in ADSCs but increased remarkably from days 14 to 28 compared with CREBBP mRNA expression (Fig. 1g). After transfection with a circCREBBP-overexpressing plasmid and a blank plasmid, chondrogenic differentiation of ADSCs was induced (Supplementary Fig. 1c). On day 21, the volume of chondrocyte microspheres detected in the overexpressed group was smaller than that in the control group with fluorescence microscopy (Supplementary Fig. 1d), and Alcian blue staining suggested a lower amount of extracellular matrix (Fig. 1h). Collectively, these results revealed the critical circular structure of circCREBBP, which may be related to degeneration in the late stage of ADSC chondrogenesis.

### circCREBBP is present at relatively high levels in osteoarthritic cartilage and induces matrix degradation in human chondrocytes

**Fig. 2:**
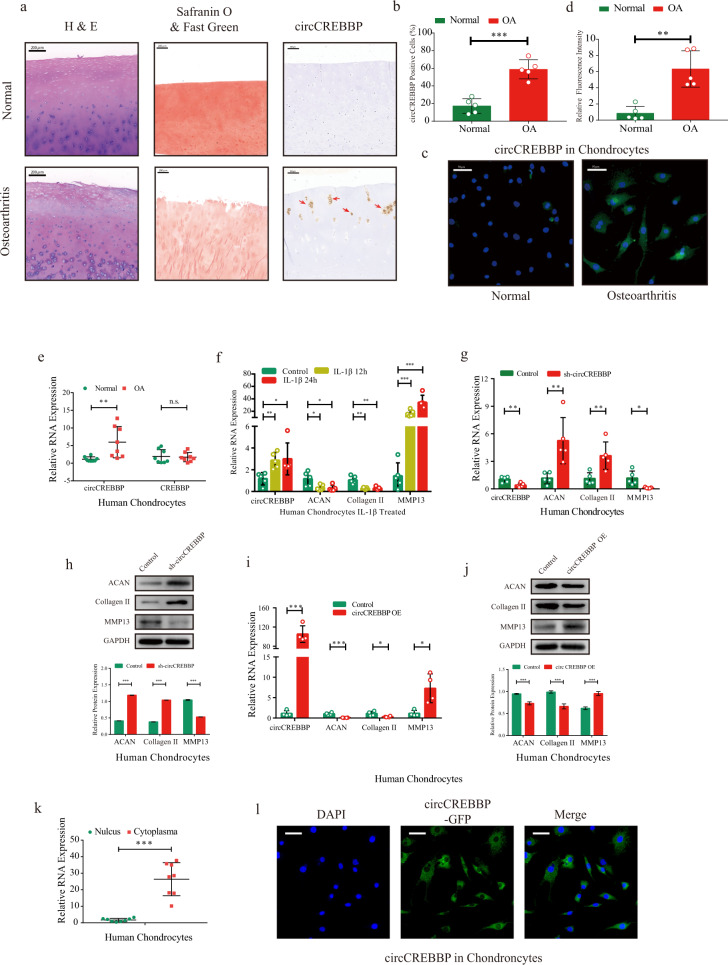
circCREBBP induces matrix degradation in human chondrocytes. **a** Hematoxylin–eosin staining (scale bar, 200 μm), safranin-O/fast green staining (scale bar, 200 μm), and in situ hybridization of circCREBBP-labeled (scale bar, 100 μm) cartilage tissues from healthy controls or OA patients. **b** Quantitative analysis of circCREBBP expression in the cartilage with ISH (*n* = 5). **c** circCREBBP RNA-FISH staining of normal or osteoarthritic chondrocytes. Scale bars: 200 μm. **d** Quantification of circCREBBP RNA-FISH analysis (*n* = 5). **e** Relative circCREBBP and CREBBP RNA expression was validated by RT‒qPCR in normal or osteoarthritic chondrocytes (*n* = 8). **f** The expression of circCREBBP, ACAN, Collagen II, and MMP13 in human chondrocytes stimulated with IL-1β (10 ng/mL) for 12 or 24 h (*n* = 5). After 48 h of transfection of shRNA-circCREBBP or vector, the mRNA and protein expression of circCREBBP, ACAN, Collagen II and MMP13 were measured by RT‒qPCR (**g**) and western blot with quantitative analysis (**h**) in human chondrocytes (*n* = 5). The mRNA (**i**) and protein (**j**) levels of circCREBBP, ACAN, Collagen II, and MMP13 in the circCREBBP overexpression human chondrocytes (*n* = 4). **k** Nuclear and cytoplasmic RNAs were extracted from human chondrocytes, and RT‒qPCR was used for circCREBBP detection (*n* = 8). **l** FISH with circCREBBP probes revealed cellular localization. Nuclei were stained with DAPI. Scale bar: 50 μm. GAPDH was used as the internal reference. Two-tailed Student’s *t* test or one-way analyses of variance were used for a significance level, and Dunnett’s test was used to perform multiple comparisons. Each bar represents the mean ± SD. *P* < 0.05, *P* < 0.01, *P* < 0.001, n.s. no significance.

To clarify the roles of circCREBBP in chondrocyte degeneration, we collected eight human osteoarthritic cartilage samples and eight normal control cartilage samples to detect circCREBBP expression (Fig. 2a). Quantitative analysis for in situ hybridization with the circCREBBP probe revealed higher expression of circCREBBP in the osteoarthritic tissues than in the normal tissues (Fig. 2b). Similarly, we isolated chondrocytes from the cartilage and observed higher expression levels of circCREBBP in the osteoarthritic chondrocytes using RNA fluorescence in situ hybridization (FISH) (Fig. 2c, d). Moreover, divergent primer tests for cDNA and genomic DNA, Sanger sequencing, and resistance to RNase R digestion experiments were conducted to verify the specific circular biological structure in chondrocytes (Supplementary Fig. 1e–h). RT‒qPCR data from the OA and normal groups indicated that circCREBBP was upregulated in osteoarthritic chondrocytes, unlike the CREBBP linear transcript without differential expression (Fig. 2e). RT‒qPCR to detect *ACAN*, *Collagen II*, and *MMP13* confirmed the degenerative state in the IL-1β-treated chondrocytes and the relatively high level of circCREBBP (Fig. 2f).

To assess the effect of circCREBBP on the regulation of chondrocyte degeneration, we generated circCREBBP small interfering RNAs (siRNAs) for gene silencing experiments. Si-circCREBBP#1 showed the highest efficiency for circCREBBP knockdown by RT‒qPCR and did not affect linear transcripts (Supplementary Fig. 2a). Western blotting analyses of the chondrogenic markers aggrecan and Collagen II and the degenerative marker MMP13 indicated that circCREBBP knockdown improved the anabolism of ECM in human chondrocytes (Supplementary Fig. 2b). For further functional experiments, we constructed a short hairpin RNA (shRNA) plasmid based on si-circCREBBP#1′ sequences. After transfection of sh-circCREBBP in chondrocytes (Fig. 2g, h) and SW1353 cells (Supplementary Fig. 2c, d), the mRNA and protein expression of aggrecan and Collagen II was upregulated, whereas MMP13 expression was downregulated. In contrast, RT‒qPCR data and western blotting analysis both confirmed that overexpression of circCREBBP in human chondrocytes (Fig. 2i, j) and SW1353 cells (Supplementary Fig. 2e, f) increased MMP13 expression but reduced aggrecan and Collagen II mRNA and protein levels. Similar trends in aggrecan and MMP13 expression were observed using IF in human chondrocytes (Supplementary Fig. 2g).

Furthermore, nuclear and cytoplasmic fractionation assays coupled with RT‒qPCR (Fig. 2k) and FISH (Fig. 2l) confirmed that circCREBBP was located predominantly in the cytoplasm.

### miR-1208 triggers TGFβ2 expression and improves the ECM in chondrocytes

**Fig. 3:**
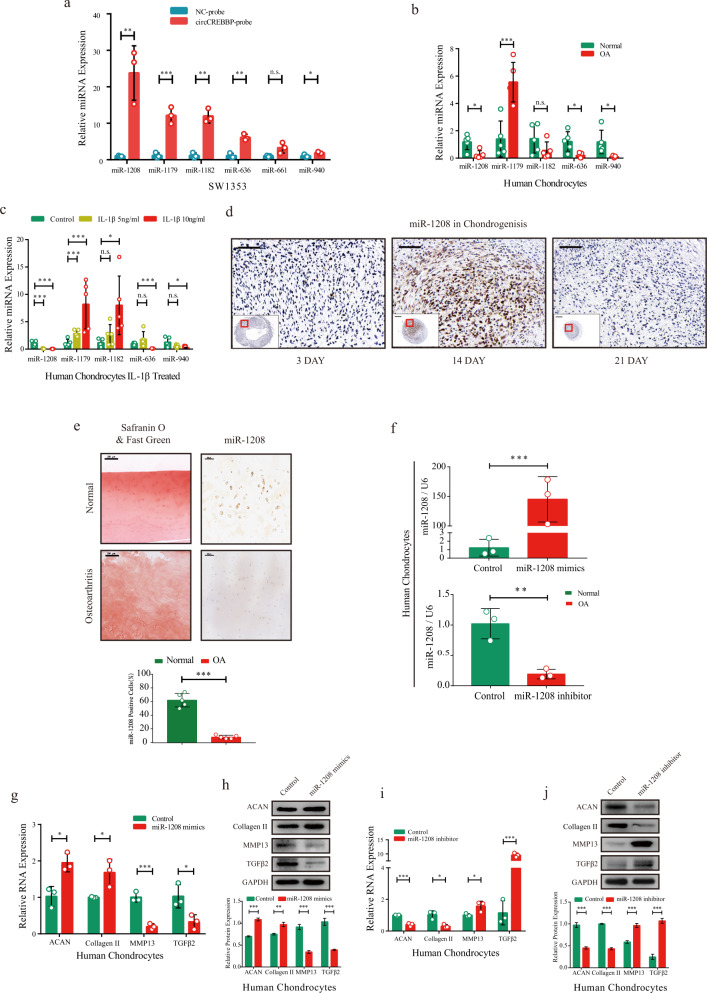
Targeting miR-1208 affects matrix-degrading and anabolic factors in human chondrocytes. **a** Multiple miRNA candidates were pulled down by circCREBBP probe and examined by RT‒qPCR (*n* = 3). RT‒qPCR revealed the expression of miRNA candidates in normal and osteoarthritic chondrocytes (**b**), followed by stimulation with different doses of IL-1β for 24 h (**c**) (*n* = 5). **d** In situ hybridization of miR-1208-labeled in the pathological section of microspheres during chondrogenesis. Scale bar, 200 μm. **e** Quantitative analysis of miR-1208 expression in osteoarthritic and normal cartilage with ISH. Scale bar, 100 μm (*n* = 5). **f** The efficiency of the miR-1208 mimics and miR-1208 inhibitor was qualified with RT‒qPCR in human chondrocytes (*n* = 3). The changes in the mRNA and protein expression of ACAN, Collagen II, MMP13, and TGFβ2 in human chondrocytes were evaluated by RT‒qPCR (**g**) and western blots with quantitative analysis (**h**) (*n* = 3). RT‒qPCR (**i**) and western blots with quantitative analysis (**j**) of the treatment effect of the miR-1208 inhibitor were obtained from human chondrocytes (*n* = 3). GAPDH or U6 was used as the internal reference. Two-tailed Student’s *t* test or one-way analyses of variance were used for a significance level, and Dunnett’s test was used to perform multiple comparisons. Each bar represents the mean ± SD. *P* < 0.05, *P* < 0.01, *P* < 0.001, n.s. no significance.

As circCREBBP with multiple Ago2-binding sites was abundant in the chondrocytic cytoplasm, we proposed a competing endogenous RNA regulatory mechanism of circCREBBP for further analysis. As shown in Fig. 3a, the specific enrichment of miR-1208, miR-1179, miR-1182, miR-636, and miR-940 (five of the highest scored miRNAs in the CircleInteractome database) detected in the circCREBBP pulldown pellet was significantly greater than that in the control group. Subsequently, RT‒qPCR analysis of both chondrocytes with or without OA (Fig. 3b) and human chondrocytes with or without stimulation of IL-1β (Fig. 3c) revealed a protective role for miR-1208, which showed the opposite function of circCREBBP. In situ hybridization of miR-1208 in chondrogenic microspheres suggested that its expression peaked on day 14 during ADSC chondrogenesis (Fig. 3d). The miR-1208 expression levels in osteoarthritic cartilage sections quantified by the percentage of positive cells were significantly lower than those in normal cartilage (Fig. 3e).

TGFβ2, as the predicted downstream target of miR-1208 in TargetScan (http://www.targetscan.org/) and miRTarBase (http://mirtarbase.mbc.nctu.edu.tw/), was evaluated along with ECM metabolic markers. Overexpression of miR-1208 in human chondrocytes (Fig. 3f) led to increased aggrecan and Collagen II mRNA (Fig. 3g) and protein (Fig. 3h) levels but weakened MMP13 and TGFβ2 expression. In contrast, inhibition of miR-1208 resulted in a significant increase in MMP13 and TGFβ2 expression and a corresponding decrease in aggrecan and Collagen II according to RT‒qPCR (Fig. 3i) and western blotting with quantitative analyses (Fig. 3j). The same test in SW1353 cells (Supplementary Fig. 3a–f) and IF analyses (Supplementary Fig. 2g) in chondrocytes for aggrecan, MMP13, and TGFβ2 expression revealed the obvious effects of miR-1208 overexpression or knockdown. These results indicate that miR-1208 negatively regulates TGFβ2 and alleviates chondrocyte ECM degradation.

### circCREBBP sponges miR-1208 in human chondrocytes

**Fig. 4:**
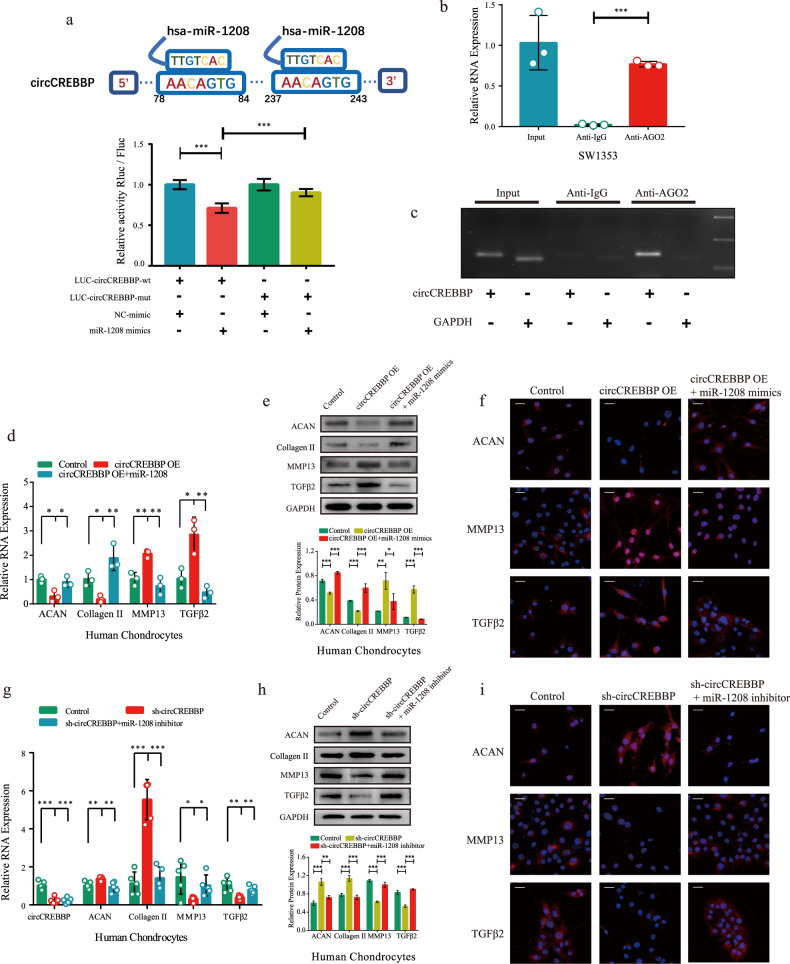
circCREBBP serves as a sponge of miR-1208. **a** Schematic illustration showing the predicted binding sites in circCREBBP for miR-1208. Luciferase activity in SW1353 cells cotransfected with a dual-luciferase reporter vector containing the wild-type or mutant circCREBBP sequence and the miR-1208 mimics or control (*n* = 3). The circCREBBP level in SW1353 cells was detected by an AGO2 RNA immunoprecipitation assay (RIP assay) with RT‒qPCR (**b**) and agarose gel electrophoresis (**c**). IgG was used as a control, and GAPDH was used as a negative control (*n* = 3). After 48 h of cotransfection with circCREBBP and miR-1208 mimics or mimic-control in human chondrocytes, the mRNA expression of ACAN, Collagen II, MMP13, and TGFβ2 was examined by RT‒qPCR (**d**). Protein levels of ACAN, Collagen II, MMP13, and TGFβ2 were tested by western blotting with quantitative analysis (**e**) and IF (**f**). Chondrocytes were coinfected with sh-circCREBBP and miR-1208 inhibitor or inhibitor control for 48 h and were evaluated for mRNA levels of ACAN, Collagen II, MMP13, and TGFβ2 with RT‒qPCR (**g**) and protein levels with western blotting (**h**) and IF (**i**). GAPDH was used as the internal reference. Two-tailed Student’s *t* test was used for the significance level. Each bar represents the mean ± SD. *P* < 0.05, *P* < 0.01, *P* < 0.001, n.s. no significance.

To confirm the direct binding between circCREBBP and miR-1208, we constructed a circCREBBP luciferase plasmid (wild-type [WT]) and a mutant plasmid and then cotransfected HEK293T cells with a miR-1208 mimic or NC mimic. Dual-luciferase assays revealed that the luciferase activity of circCREBBP-WT was notably reduced by miR-1208, whereas no obvious change was found after mutating the predicted binding site in circCREBBP (Fig. 4a). Moreover, an Ago2 RNA immunoprecipitation (Ago2-RIP) assay was performed in SW1353 cells, which validated circCREBBP binding to the Ago2 protein (Fig. 4b, c).

To determine the regulatory relationship between circCREBBP and miR-1208, we conducted rescue experiments in human chondrocytes. RNA expression levels were detected by RT‒qPCR, and protein levels were quantified using western blotting and IF analysis. Notably, overexpression of miR-1208 alleviated ECM catabolism and upregulation of TGFβ2 induced by circCREBBP-OE (overexpression) transfection in chondrocytes (Fig. 4d–f). miR-1208 inhibition rescued ECM anabolism and the downregulation of TGFβ2 induced by circCREBBP knockdown (Fig. 4g–i). These results indicate that circCREBBP can function as a competing endogenous RNA to regulate target TGFβ2 expression and chondrocyte indicators by sponging miR-1208.

### TGFβ2 drives OA progression in vitro and in vivo

**Fig. 5:**
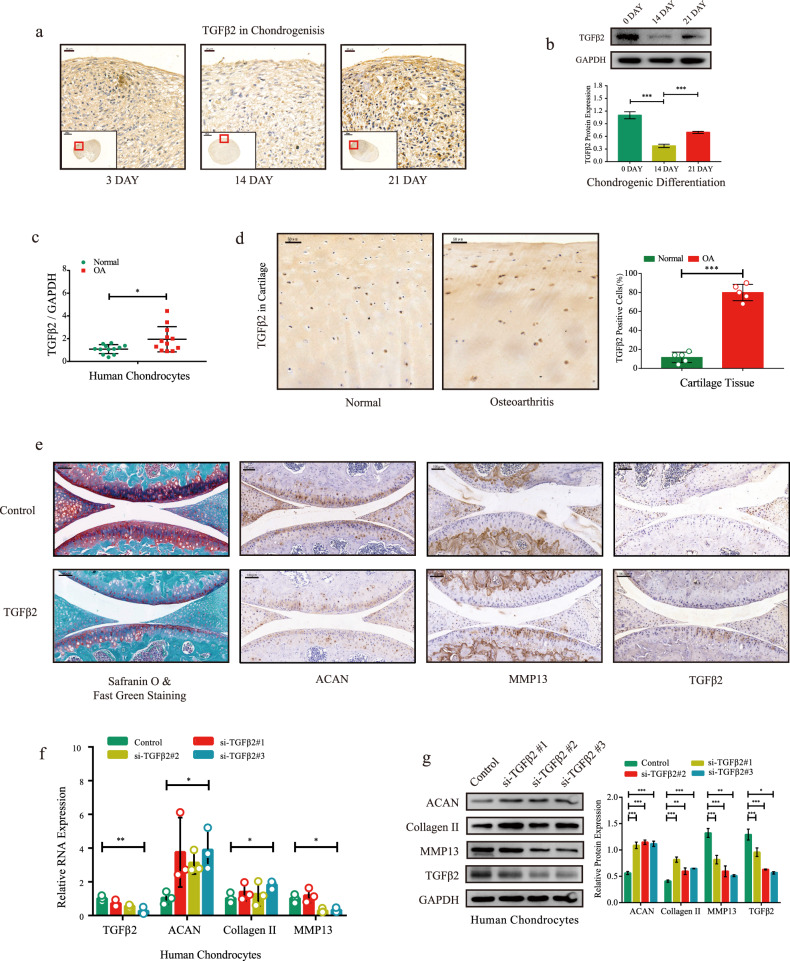
TGFβ2 triggers matrix degradation in vitro and in vivo. **a** Immunohistochemistry analysis of TGFβ2 was performed in the pathological sections of microspheres during chondrogenesis. Scale bar, 200 μm. **b** Western blotting with quantitative analysis showing the protein level of TGFβ2 during chondrogenesis (*n* = 3). TGFβ2 expression levels in normal and osteoarthritic cartilage were assessed by RT‒qPCR (**c**, *n* = 12) and IHC (**d**) (*n* = 5 ). **e** Safranin-O & fast green staining and immunohistochemistry analysis (ACAN, Collagen II, MMP13, and TGFβ2) of the knee joint in 10-week-old mice with intra-articular injection of TGFβ2 or vector. Scale bar: 100 μm. After transfection of siRNA-TGFβ2 in human chondrocytes for 48 h, the expression levels of ACAN, Collagen II, MMP13, and TGFβ2 were evaluated by RT‒qPCR (**f**) and western blots with quantitative analysis (**g**) (*n* = 3). GAPDH was used as the internal reference. Two-tailed Student’s *t* test was used for the significance level. Each bar represents the mean ± SD. *P* < 0.05, *P* < 0.01, *P* < 0.001, n.s. no significance.

To evaluate whether TGFβ2 is the downstream target of the circCREBBP/miR-1208 axis, we assessed TGFβ2 expression during chondrogenesis (days 3, 14, and 21) using immunohistochemistry (IHC) staining (Fig. 5a) and western blotting (Fig. 5b) in chondrogenic microspheres. C57B/L6J mice were isolated at 16.5 days post-coitum (E16.5) and subjected to in situ hybridization analyses for circCrebbp, miR-1208, and TGFβ2 (Supplementary Fig. 4a). Importantly, although moderate to high levels of miR-1208 expression were detected in proliferating chondrocytes, little to no miR-1208 expression was observed in hypertrophic chondrocytes. In contrast, circCrebbp and TGFβ2 expression were detected in hypertrophic chondrocytes, with little to no expression observed in proliferating chondrocytes.

To determine the effect of TGFβ2 on chondrocyte degeneration and ECM catabolism, we performed RT‒qPCR (Fig. 5c) and IHC with quantitative analysis (Fig. 5d) to detect TGFβ2 mRNA and protein expression in 12 pairs of osteoarthritic and normal cartilage samples. We found that TGFβ2 was highly expressed in the osteoarthritic cartilage. Furthermore, in human chondrocytes collected from five patients with OA, stimulation with TGFβ2 dose- and time-dependently decreased aggrecan and Collagen II expression and increased MMP13 expression (Supplementary Fig. 4b). We used TGFβ2 at 5 ng/mL for 48 h as the standard dose and time for the follow-up study. Upregulated MMP13 and TGFβ2 and downregulated aggrecan were validated by IF analysis in human chondrocytes stimulated with TGFβ2 (Supplementary Fig. 4c).

To examine the impact of cartilage exposure to TGFβ2 in vivo, we injected TGFβ2 into mouse knee joints once per week and harvested the joint samples 1 week after triple injection. Multiple intra-articular injections of TGFβ2 induced OA-like changes in the knee joint, as visualized by safranin-O and fast green staining and IHC staining for aggrecan, MMP13, and TGFβ2 (Fig. 5e). Furthermore, transfection of TGFβ2 siRNAs enhanced aggrecan and Collagen II expression and inhibited MMP13 expression, as detected by RT‒qPCR (Fig. 5f) and western blotting analysis (Fig. 5g).

### circCREBBP upregulates TGFβ2 to activate the ALK1/Smad1/5 signaling pathway in human chondrocytes

**Fig. 6:**
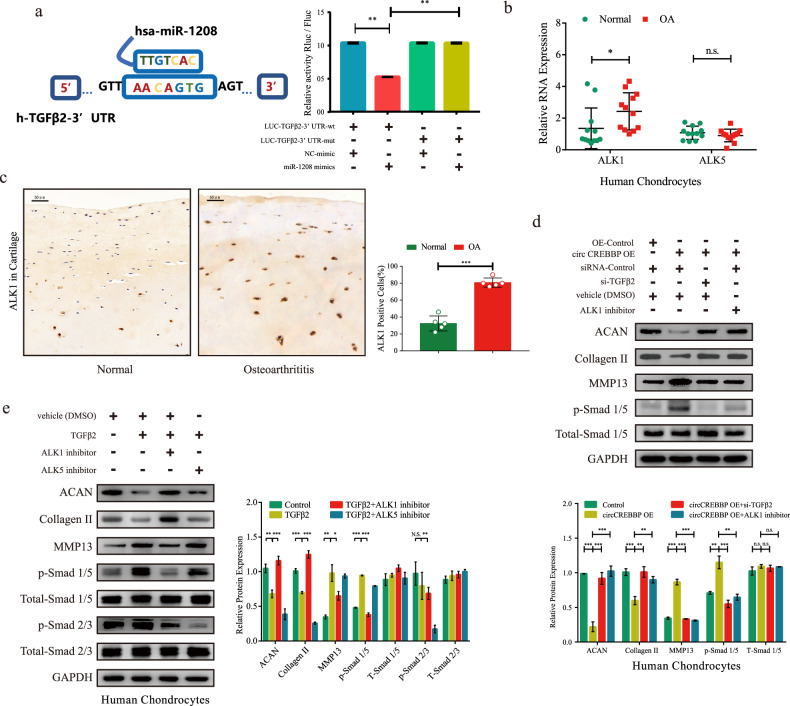
The TGFβ2/Smad1/5 pathway is the downstream target of the circCREBBP/miR-1208 axis. **a** Schematic illustration showing the predicted binding sites in the TGFβ2-3′ UTR for miR-1208. Luciferase activity in SW1353 cells cotransfected with a dual-luciferase reporter vector containing the wild-type or mutant TGFβ2-3′ UTR sequence and the miR-1208 mimics or control (*n* = 3). The expression levels of ALK1 and ALK5 in normal and osteoarthritic chondrocytes were tested by RT‒qPCR (**b**, *n* = 12), and IHC analysis for ALK1 antibodies was performed in cartilage tissues (**c**, *n* = 5). Scale bar, 50 μm. **d** The protein levels of ACAN, Collagen II, and MMP13 and the phosphorylation levels of TGFβ2/Smad pathway members in human chondrocytes coinfected with 5 ng/mL TGFβ2 recombinant protein and either ALK1 inhibitor (K02288, 0.5 µM) or ALK5 inhibitor (SB-505124, 1 μM) were quantified by western blotting (*n* = 3). **e** After cotransfection of circCREBBP or OE-control, si-TGFβ2 or si-control, and 0.5 µM ALK1 inhibitor or vector (DMSO) in human chondrocytes, ACAN, Collagen II, and MMP13 expression and phosphorylation levels of Smad1/5 were quantified by western blotting (*n* = 3). GAPDH was used as the internal reference. Two-tailed Student’s *t* test was used for the significance level. Each bar represents the mean ± SD. *P* < 0.05, *P* < 0.01, *P* < 0.001, n.s. no significance.

The binding sites of miR-1208 to the TGFβ2 3′ untranslated region (UTR) are indicated in Fig. 6a. The dual-luciferase assay showed that cotransfection of miR-1208 mimic and TGFβ2-WT decreased luciferase activity, whereas cotransfection of miR-1208 mimic and TGFβ2 with the mutated miR-1208 binding site did not change luciferase activity.

To clarify the mechanism of TGFβ2 in chondrocytes regulated by circCREBBP/miR-1208, we assessed the possible receptors in the TGFβ/Smad signaling pathway. ALK1 exhibited high mRNA (Fig. 6b) and protein (Fig. 6c) levels in osteoarthritic cartilage compared to normal cartilage, whereas ALK5 expression was not significantly different. Moreover, western blotting with quantitative analysis revealed that ALK1 knockdown inhibited Smad1/5 phosphorylation and alleviated ECM catabolism in human chondrocytes (Fig. 6d) and SW1353 cells (Supplementary Fig. 4d) stimulated by TGFβ2. Importantly, both TGFβ2 knockdown and ALK1 inhibition reversed circCREBBP-OE-induced Smad1/5 phosphorylation and ECM catabolism in human chondrocytes (Fig. 6e) and SW1353 cells (Supplementary Fig. 4e). This result indicates that circCREBBP activated the TGFβ/Smad signaling pathway by increasing TGFβ2 interactions with ALK1.

### Injection of sh-circCrebbp alleviates OA in the mouse model

To gain further insight into the impact of circCREBBP in vivo, we intra-articularly administered sh-circCrebbp AAV to a DMM mouse model with or without TGFβ2 injection. The specific AAV efficiently infected the cartilage and surrounding tissues, as observed by the detection of green fluorescent protein (Supplementary Fig. 4f). Knee joints from the AAV-sh-circCrebbp- and AAV-sh-control-treated mice were harvested and analyzed histologically (Supplementary Fig. 4g).

**Fig. 7:**
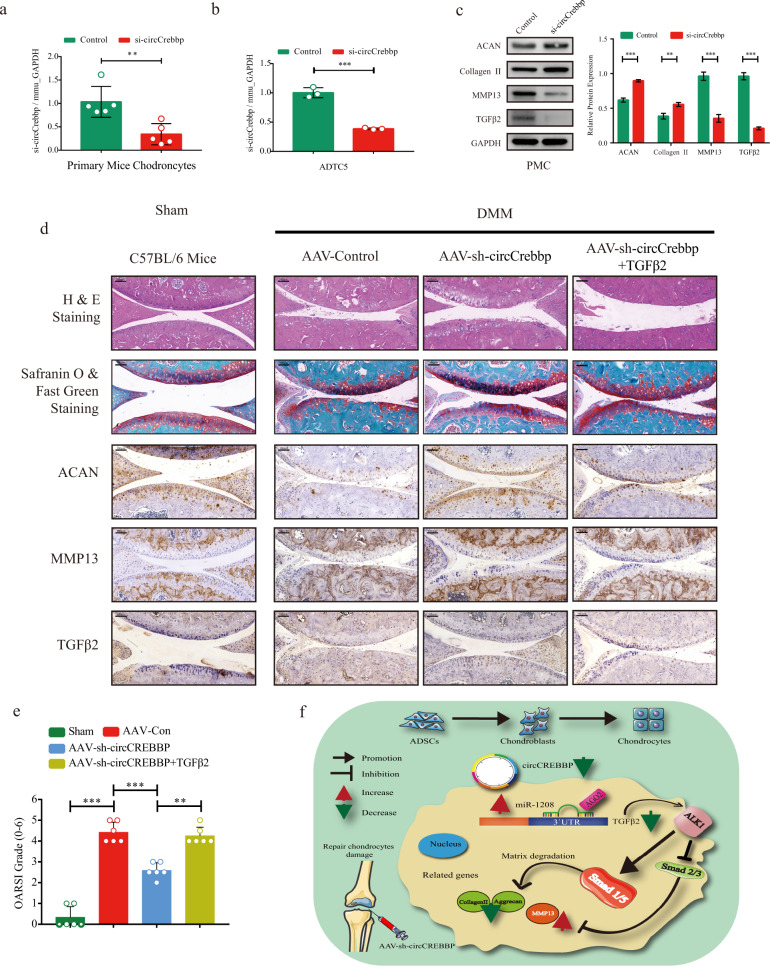
mmu_circCrebbp and mmu_TGFβ2 affect cartilage matrix degradation in vivo. Validation of circCrebbp knockdown using siRNA was verified by RT‒qPCR in primary mouse chondrocytes (**a**, *n* = 5) and ADTC5 cells (**b**, *n* = 3). **c** Protein levels of ACAN, Collagen II, MMP13, and TGFβ2 were quantified by western blots in primary mouse chondrocytes transfected with si-circCrebbp (*n* = 3). **d** Representative images of H&E staining, safranin-O/fast green staining, and IHC staining (ACAN, Collagen II, MMP13, and TGFβ2) were determined in cartilage tissues from the sham group and three groups after DMM: AAV-control, AAV-sh-circCrebbp, and AAV-shRNA+TGFβ2. Scale bar, 100 μm. **e** OARSI scores of the knee joints of DMM mice from four groups (*n* = 6). **f** Graphical abstract of the present research. GAPDH was used as the internal reference. Two-tailed Student’s *t* test was used for the significance level. Each bar represents the mean ± SD. *P* < 0.05, *P* < 0.01, *P* < 0.001, n.s. no significance.

Considering that circCREBBP is conserved between humans and mice, we assessed the effects of circCREBBP knockdown in primary mouse chondrocytes and ATDC5 cells using RT‒qPCR (Fig. 7a, b). After si-circCrebbp transfection in primary mouse chondrocytes, aggrecan, and Collagen II protein expression was upregulated, whereas MMP13 and TGFβ2 protein levels were decreased (Fig. 7c).

Hematoxylin and eosin staining, safranin-O and fast green staining, and IHC analysis of aggrecan, MMP13, and TGFβ2 were performed to evaluate cartilage degradation. As shown in Fig. 7d, AAV-sh-circCrebbp intra-articular injection relieved the degenerative changes in the cartilage of the mice with DMM-induced OA, which revealed lower Osteoarthritis Research Society International (OARSI) scores of the knee joints than those in the control group (Fig. 7e). Interestingly, restoration of cartilage degeneration was not observed when AAV-sh-circCrebbp was injected simultaneously with TGFβ2.

Taken together, our results demonstrate the clinical potential of circular RNA circCREBBP and reveal its role in chondrocyte ECM catabolism and the progression of OA. Cartilage degeneration is induced upon circCREBBP sponging in the miR-1208/TGFβ2-Smad1/5 pathway (Fig. 7f). Inhibition of circCREBBP shows treatment potential in patients with OA.

## Discussion

Osteoarthritis is thought to be the most prevalent age-related chronic joint disease, with pain and loss of function as the main features^4,8^. Despite efforts in recent decades to clarify the molecular mechanisms underlying this disease, effective disease-modifying drugs are lacking^6,34^. Chondrocytes are the only resident cell type in cartilage and are stimulated by cytokines and growth factors to undergo catabolic and abnormal differentiation, leading to ECM degradation^5,7^.

Recently, the regulatory potential of circRNAs in OA has attracted increased attention^35^. For example, studies have shown that circSERPINE2 alleviates chondrocyte apoptosis and promotes anabolism of the ECM through the mir1271-5p/ERG pathway^36^; the ciRS-7/miR-7 axis ameliorates cartilage degradation and inhibits autophagy via PI3K/AKT/mTOR activation^37^; circPDE4D regulates glycosaminoglycan composition and inhibits matrix degeneration enzymes^38^; and circ0083429 regulates the ECM by downregulating miR-346/Smad3 in chondrocytes^39^. According to our previous study, the chondrogenic differentiation of MSCs started 7 days after induction, with cartilage matrix gradually synthesized, reaching the peak of the expression of Collagen II and Aggrecan at days 14–21, while matrix metalloproteinases (MMPs) and indicators of chondrocyte hypertrophy increased rapidly. At the late stage of differentiation, from approximately the 21st day to the 28th day, cartilage matrix components were significantly downregulated, and MMPs were significantly increased^40–42^. In this study, we demonstrated that circCREBBP is temporarily downregulated on day 14 during chondrogenic differentiation of ADSCs, which is the flourishing stage of matrix synthesis. We hypothesized that circCREBBP is a critical factor that blocks ECM synthesis and causes cartilage degeneration. Our experiments revealed circCREBBP overexpression in the chondrocytes of osteoarthritic cartilage and the IL-1β-induced OA model, indicating that this circRNA is associated with the development and progression of OA. Therefore, gain- and loss-of-function assays were designed to validate the role of circCREBBP in chondrocyte degeneration.

CircCREBBP originates from the backsplicing of exons of *CREBBP*, which has histone acetyltransferase activity^43^. In this study, even under RNase R treatment and actinomycin D addition, circCREBBP was highly stable as a circular RNA, as described previously^44^. The cytoplasmic location and abundant Ago2-binding sites predicted by the CircInteractome database indicated that circCREBBP functions as a sponge for miRNA. Further experiments showed that these six miRNAs were captured by a specific circCREBBP probe, but only miR-1208 was expressed at low levels in both osteoarthritic chondrocytes and IL-1β-induced chondrocytes. Further analysis indicated that circCREBBP is a sponge for miR-1208.

The best-characterized signaling pathway downstream of TGFβs is the canonical Smad2/3-dependent pathway, which promotes the anabolic activity of the ECM^24,45^. A previous study suggested that TGFβ-Smad2/3 signaling pathways promote SOX9-dependent transcriptional activity by facilitating the recruitment of CREBBP to the *Collagen II* enhancer^46^. Thus, CREBBP participates in accelerating proteoglycan synthesis and *Collagen II* expression. However, circCREBBP expression, rather than linear CREBBP expression, was highly upregulated in the late stage of chondrogenesis in ADSCs, chondrocytes from patients with OA, and IL-1β-induced chondrocytes. Moreover, knockdown of circCREBBP by siRNAs with specific sequences did not affect linear CREBBP transcription or the promotion of *Collagen II* and *ACAN* expression, indicating an independent role for circCREBBP in ECM catabolism and cartilage degradation. TGFβs can also induce phosphorylation of Smad1/5, and ALK1 signaling (via the Smad1/5 route) results in chondrocyte responses markedly different from those induced by ALK5 (via Smad2/3) signaling^47^. Although age, ALK5 kinase activity, the ALK1/ALK5 ratio, and endoglin are considered switches for signaling pathways, the exhaustive mechanisms and their functional relevance to cartilage degradation are unclear^27,47–50^. In our study, both TGFβ2 and ALK1 were downregulated on day 14 during ADSC-induced chondrogenesis but upregulated in osteoarthritic cartilage compared to normal cartilage. Subsequently, TGFβ2 (miR-1208 target) was confirmed to aggravate ECM degradation and cartilage degeneration in vivo and in vitro via phosphorylation of Smad1/5 rather than Smad2/3. Notably, the addition of TGFβ2 siRNA or ALK1 inhibitor counteracted the increased catabolism in the ECM by circCREBBP overexpression. Therefore, we proposed a competing endogenous RNA network formed by circCREBBP acting as a miR-1208 sponge to promote TGFβ2 expression, leading to Smad1/3 phosphorylation and enhanced ECM catabolism, thereby contributing to the progression of OA.

Intra-articular injection has the advantages of direct targeting, which avoids systemic side effects and thus provides an attractive route for molecular therapy to treat OA^51,52^. We confirmed that injection of AAV-sh-circCrebbp recovers DMM-induced cartilage degradation by regulating ECM-related genes (*Collagen II*, *ACAN*, and *MMP13*), but this positive effect was inhibited by the addition of TGFβ2.

Taken together, our findings highlight a subversive role of circCREBBP in the pathogenesis of OA and show that targeting the circCREBBP-miR-1208-TGFβ2 axis is a promising approach for OA treatment.

## Supplementary information

Supplementary Materials
