# Author Correction: Circular RNA CREBBP modulates cartilage degradation by activating the Smad1/5 pathway through the TGFβ2/ALK1 axis

**DOI:** 10.1038/s12276-023-01029-6

**Published:** 2023-06-09

**Authors:** Yiyang Xu, Guping Mao, Dianbo Long, Zengfa Deng, Ruobin Xin, Ziji Zhang, Ting Xue, Weiming Liao, Jie Xu, Yan Kang

**Affiliations:** 1 Department of Joint Surgery, The First Affiliated Hospital, Sun Yat-sen University, Guangzhou, 510080 Guangdong China; 2 Guangdong Provincial Key Laboratory of Orthopaedics and Traumatology, Guangzhou, 510080 Guangdong China; 3 Department of Orthopaedics, Fujian Provincial Hospital, Shengli Clinical Medical College, Fujian Medical University, Fuzhou, 350001 Fujian China; 4 Fujian Provincial Hospital South Branch, Center of Health Management, Shengli Clinical Medical College of Fujian Medical University, Fuzhou, China

**Keywords:** Long non-coding RNAs, Phosphorylation

Correction to: *Experimental & Molecular Medicine* 10.1038/s12276-022-00865-2, published online 12 October 2022

After online publication of this article, the authors noticed an error in the Authors contributed section:

“These authors jointly supervised this work: Yiyang Xu, Guping Mao, Dianbo Long”.

The correct statement of this article should have read as below.

“These authors contributed equally: Yiyang Xu, Guping Mao, Dianbo Long”.

The authors apologize for any inconvenience caused.

The original article has been corrected.

