# Supplementary material for: Circular RNA CREBBP modulates cartilage degradation by activating the Smad1/5 pathway through the TGFβ2/ALK1 axis
Source: Exp Mol Med. 2022 Oct 12;54(10):1727–40. doi: 10.1038/s12276-022-00865-2 (PMC9636424; doi:10.1038/s12276-022-00865-2)
Supplement: Supplementary file 1 — Supplementary Materials [file 12276_2022_865_MOESM1_ESM.pdf]

Supplementary Table 1

Gene	Primiers
hsa-gapdh-F	GCACCGTCAAGGCTGAGAAC
hsa-gapdh-R	TGGTGAAGACGCCAGTGGA
hsa-Aggrecan-F	GATGTTCCCTGCAATTACCACCTC
hsa-Aggrecan-R	TGATCTCATACCGGTCCTTCTTCTG
hsa-mmp13-F	TCCTGATGTGGGTGAATACAATG
hsa-mmp13-R	GCCATCGTGAAGTCTGGTAAAAT
hsa-CollagenII-F	CCAGATGACCTTCCTACGCC
hsa-CollagenII-R	TTCAGGGCAGTGTACGTGAAC
hsa-TGFβ2-F	CCCCGGAGGTGATTTCCATC
hsa-TGFβ2-R	GGGCGGCATGTCTATTTTGTAAA
hsa_circCREBBP-F	CAGGAGGCATGGCCAAGATT
hsa_circCREBBP-R	CTCGTAGAAGCTCCGACAGTT
hsa-CREBBP-F	ATTTTGGATCATTGTTTGACTTGGA
hsa-CREBBP-R	CTTGGCCATGCCTCCTGC
mmu_circCrebbp-F	AATGCCCTACCCTGCTCCA
mmu_circCrebbp-R	TTTCCAAGTCAAACAATGATCCAA
mmu-CollagenII-F	CCCGCCTTCCCATTATTGAC
mmu-CollagenII-R	GGGAGGACGGTTGGGTATCA
mmu-Aggrecan-F	ATTTCCACACGCTACACCCTG
mmu-Aggrecan-R	TGGATGGGGTATCTGACTGTC
mmu-MMP13-F	ATGCATTCAGCTATCCTGGCCA
mmu-MMP13-R	AAGATTGCATTTCTCGGAGCCTG
mmu-GAPDH-F	TGTGTCCGTCGTGGATCTGA
mmu-GAPDH-R	TTGCTGTTGAAGTCGCAGGAG

Supplementary Table 2

Antibodies for Western Blots	Article number and manufacturer	Working concentration
CollagenII	#ab188570, Abcam	1:1000
Aggrecan	#MABT110, Millipore Sigma	1:1000
MMP13	#ab39012, Abcam	1:2000
GAPDH	#97166, CST	1:3000
TGFβ2	#19999-1-AP, ProteinTech	1:1000
ALK1	#14745-1-AP, ProteinTech	1:1000
Smad 2/3	#8685S, CST	1:2000
Smad 1	#9743S, CST	1:2000
p-Smad 2/3	#8828S, CST	1:2000
p-Smad 1/5	#9516S, CST	1:2000
Antibodies for IF	Article number and manufacturer	Working concentration
Aggrecan	#13880-1-AP, ProteinTech	1:200
MMP13	#18165-1-AP, ProteinTech	1:200
TGFβ2	#19999-1-AP, ProteinTech	1:200
Goat anti-rabbit IgG, Alexa Fluor R555	#4413S, CST	1:200

Supplementary Table 3

si-h-hsa_circ_0007637_001	GGCCAAGATTTTGGATCAT
si-h-hsa_circ_0007637_002	GAGGCATGGCCAAGATTTT
si-h-hsa_circ_0007637_003	CCAAGATTTTGGATCATTG
hsa-miR-1208 mimic-Mature	5‘-UCACUGUUCAGACAGGCGGA-3’
hsa-miR-1208 mimic-Complementary	5‘-UCCGCCUGUCUGAACAGUGA-3’
hsa-miR-1208 inhibitor	5‘-UCCGCCUGUCUGAACAGUGA-3’
si-h-TGFB2_001	CGGAGGTGATTTCCATCTA
si-h-TGFB2_002	GTCCCAAGATTTAGAACCT
st-h-TGFB2_003	GCAGCACACTCGATATGGA
mmu_circ_0006288-si-1	GAGGCAUGACCAAGAUUUUGGdTdT
shRNA-mmu_circ_0006288-Top	AATTCGAGGCATGACCAAGATTTTGGTTCAAGAGACCAAAATCTTGGTCATGCCTCTTTTTTG
shRNA-mmu_circ_0006288-Bottom	GATCCAAAAAAGAGGCATGACCAAGATTTTGGTCTCTTGAACCAAAATCTTGGTCATGCCTCG
hsa-circ-0007637-probe	TGATCCAAAATCTTGGCCATGCCTC
mmu_circ_0006288-probe	TTGTTTCTTTGCATTGCCAGTTTCTCCTGCT
miR-1208-probe	TCCGCCTGTCTGAACAGTGA

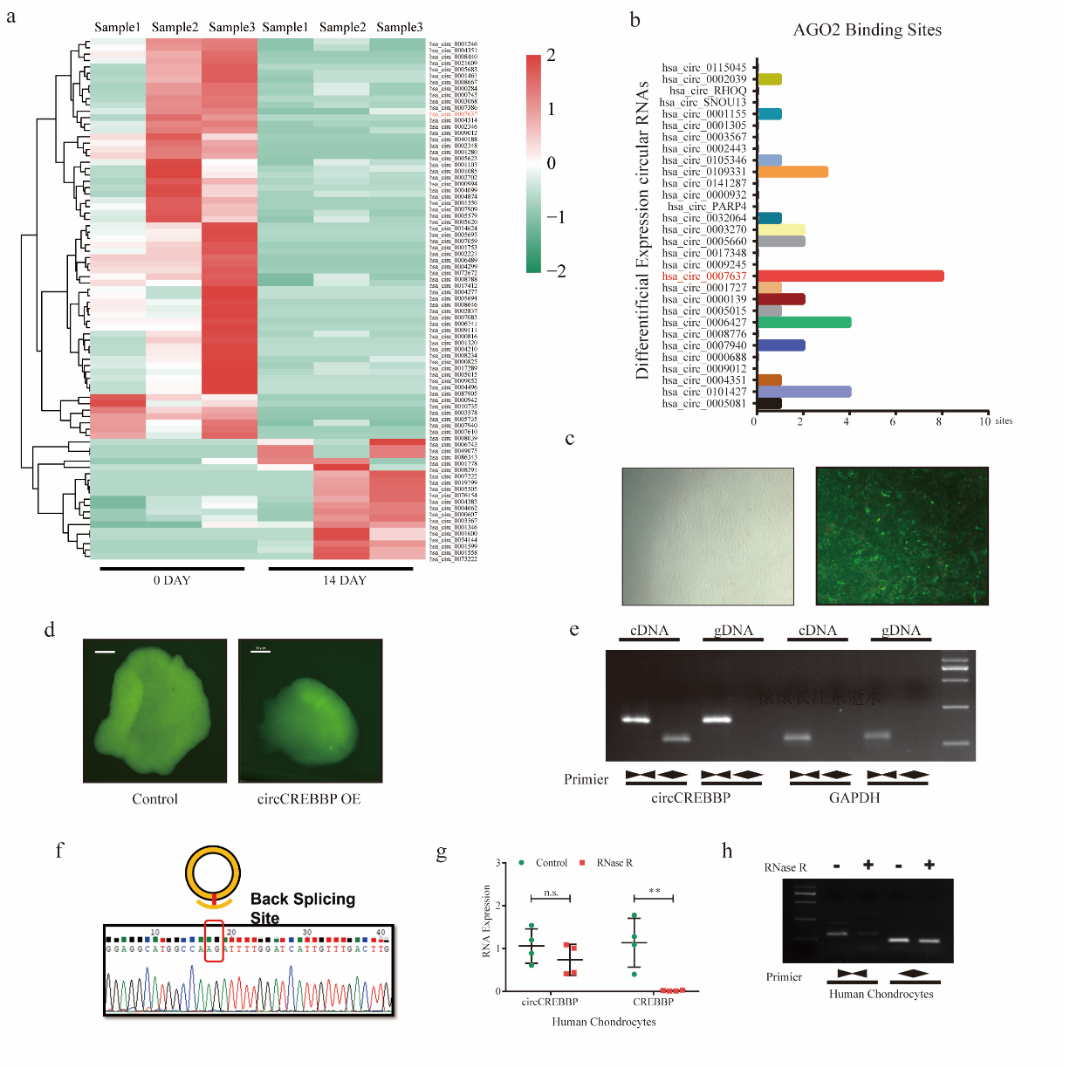

Supplementary Fig. 1

a Heat map shows significantly expressed circRNAs with ≥2-fold between day 0 and day 14 in chondrogenesis of ADSCs.

b Schematic illustration to show the number of binding sites for AGO2 in differentially expressed circRNAs, as predicted by *Circinteractome* database.

c ADSCs presented a typical morphology of spindle shake and GFP-positive cells indicated successful transfection of overexpressed or control plasmids.

d Fluorescence microscopy was used to observe chondrogenesis microspheres formed by induced ADSCs transfected with circCREBBP-OE or control plasmids.

e RT-qPCR products generated with divergent primers and linear primers in human chondrocytes showing circularization of circCREBBP. cDNA, complementary DNA. gDNA, genomic DNA.

f The presence of circCREBBP in chondrocytes was validated by Sanger sequencing. Red box represents specific head-to-tail splicing sites of circCREBBP.

g circCREBBP and CREBBP expression in in human chondrocytes treated with or without RNase R were detected by RT-qPCR. (n=4)

h Agarose gel electrophoresis assay for PCR products from divergent primers and linear primers in the ADSCs treated with or without RNase R.

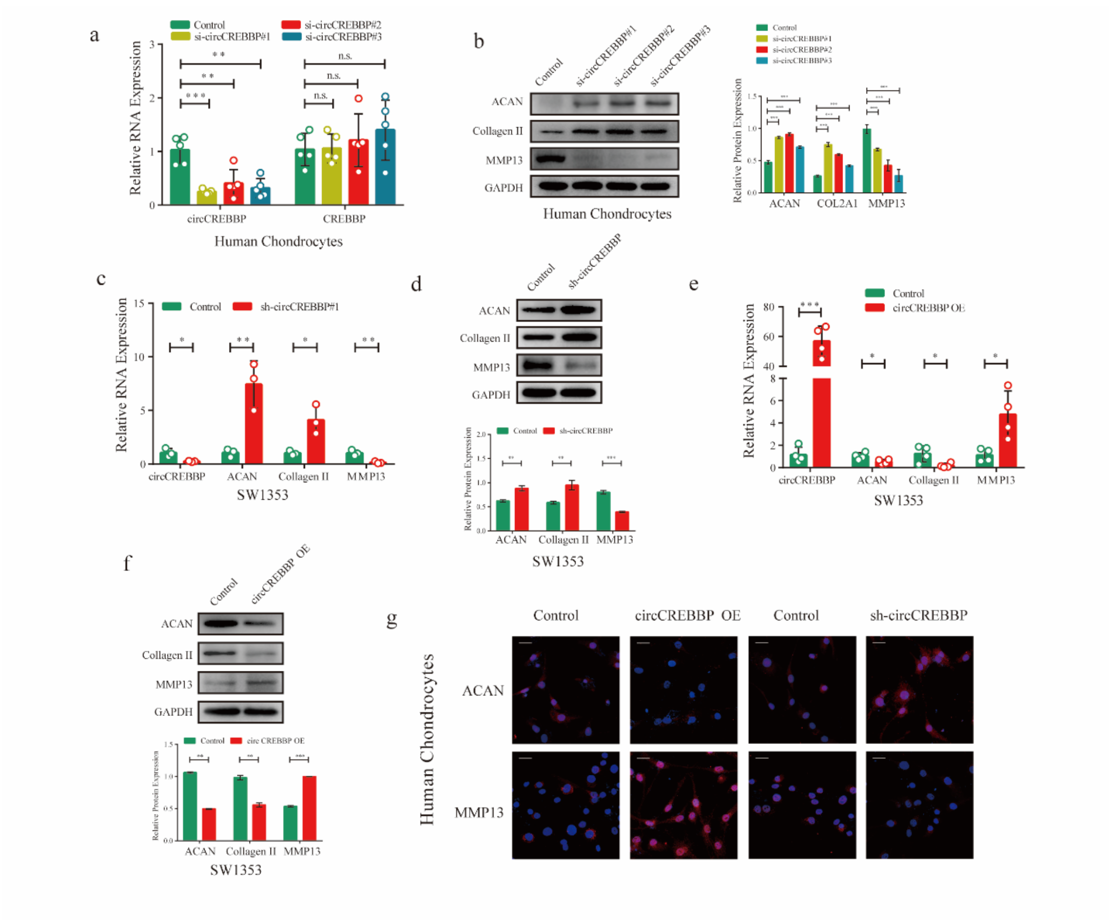

Supplementary Fig. 2

a Chondrocytes were transfected with different siRNA-circCREBBP or siRNA-control and evaluated by RT-qPCR. (n=5)

b Protein levels of ACAN, COLLAGEN II and MMP13 in human chondrocytes transfected with different siRNA-circCREBBP or siRNA-control were detected with Western blots using quantitative analysis. (n=3)

After 48 hours of transfection of shRNA-circCREBBP or vector, the mRNA and protein expression of circCREBBP, ACAN, COLLAGEN II and MMP13 were measured by RT-qPCR (c) and Western blot with quantitative analysis (d) in SW1353 cells. (n=3)

The mRNA (e) and protein (f) levels of circCREBBP, ACAN, COLLAGEN II and MMP13 in circCREBBP overexpression-treated SW1353 cells. (n=4)

g Immunofluorescence of ACAN and MMP13 in human chondrocytes infected with circCREBBP-OE or sh-circCREBBP.

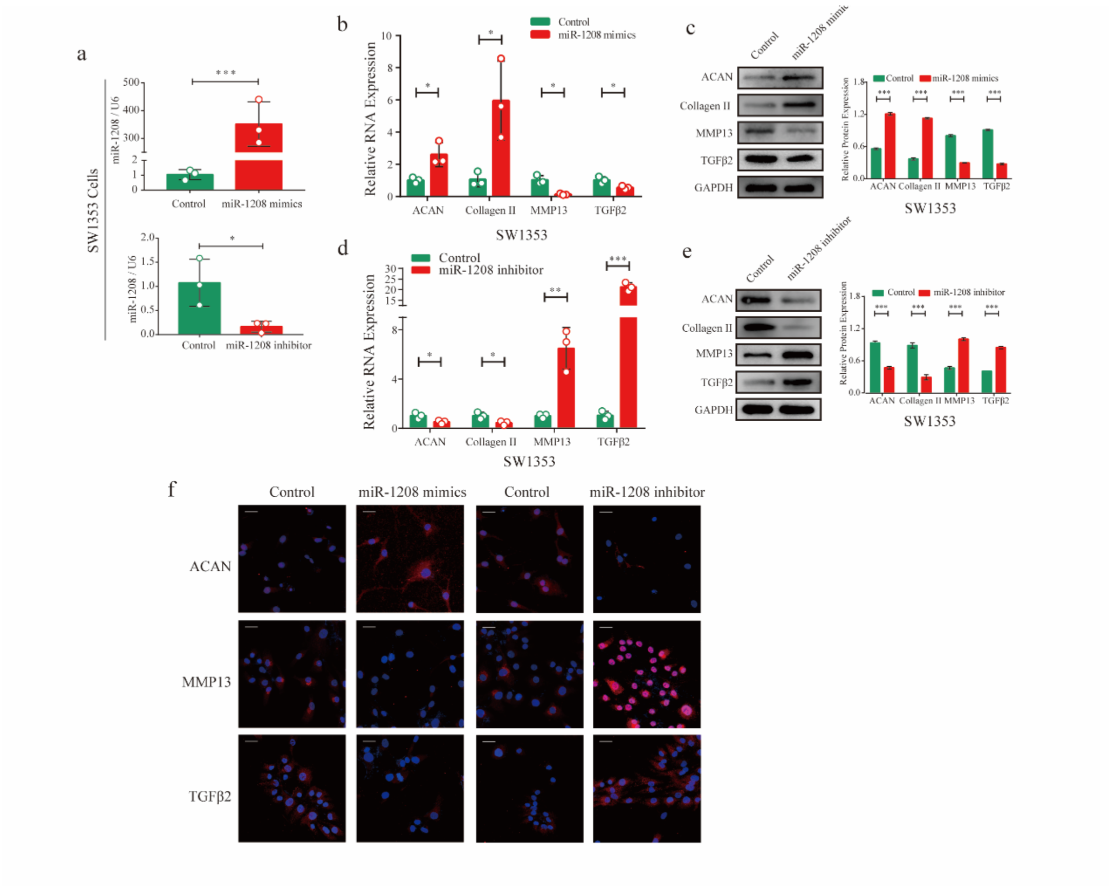

Supplementary Fig. 3

a The efficiency of miR-1208 overexpressed or knockdown was verified by RT-qPCR in SW1353 cells. (n=3)

After transfection of miR-1208 mimics or inhibitor in SW1353 cells, mRNA and protein expression of ACAN, COLLAGEN II, MMP13 and TGFβ2 were evaluated by RT-qPCR (b, d) and Wertern blots with quantitative analysis (c, e). (n=3)

f Immunofluorescence of ACAN, MMP13 and TGFβ2 in human chondrocytes after transfected with miR-1208 mimics and miR-1208 inhibitor.

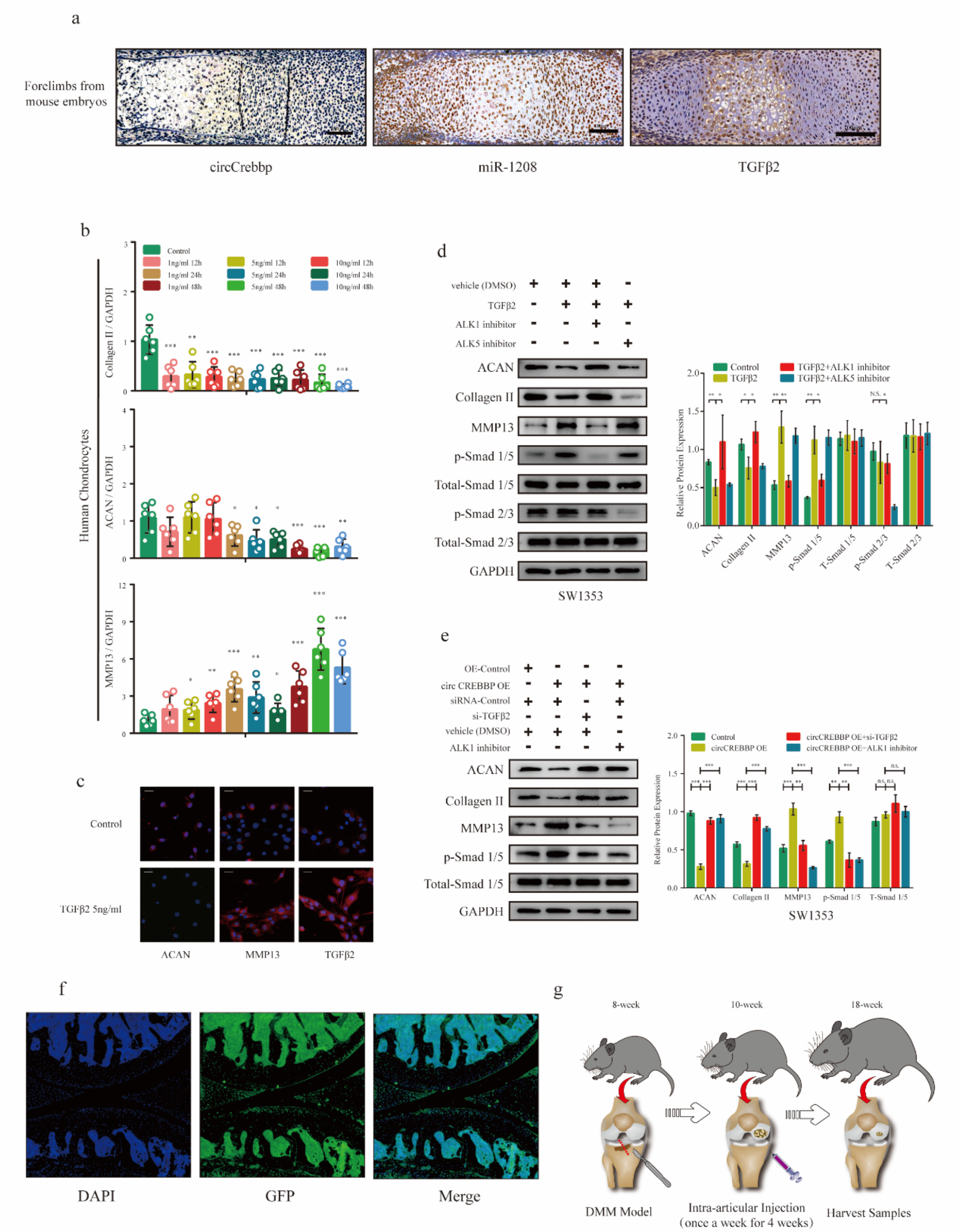

Supplementary Fig. 4

a *In situ hybridization* analysis of circCrebbp (left) and miR-1208 (middle) expression in tissue sections from the radius of mouse embryos were harvested at 16.5 days post-coitum. Sections were immunostained with antibodies specific to TGFβ2 (right).

b The mRNA levels of ACAN, COLLAGEN II and MMP13 were examined in chondrocytes with stimulation of different doses TGFβ2 after 12, 24 and 48 hours. (n=6)

c IF revealed the ACAN, MMP13 and TGFβ2 protein expression in human chondrocytes with TGFβ2 (5ng/ml) for 48 hours.

d The protein levels of ACAN, COLLAGEN II and MMP13 and phosphorylation levels of TGFβ2/Smad pathway members in sw1353 cells coinfected with TGFβ2 and either ALK1

inhibitor or ALK5 inhibitor, were quantified by Western blotting. (n=3)
e After cotransfection of circCREBBP or OE-control, si-TGFβ2 or si-control, and ALK1 inhibitor or vector (DMSO) in SW1353 cells, ACAN, COLLAGEN II and MMP13 expression and phosphorylation levels of Smad 1/5 were quantified by Western blotting. (n=3)
f Fluorescence microscopy for frozen sections confirmed that AAV-shRNA carrying GFP fragment could be taken up by chondrocytes after articular injection in mice.
g Schematic diagram illustrating the procedure of *in vivo* experiment.
